# Editorial: Uveal melanoma: from lab bench to clinic – new therapeutic advances

**DOI:** 10.3389/fmed.2023.1233214

**Published:** 2023-06-20

**Authors:** Agata Grazia D'Amico, Grazia Maugeri, Velia D'Agata, Gustav Stålhammar

**Affiliations:** ^1^Department of Drug and Health Sciences, University of Catania, Catania, Sicily, Italy; ^2^Department of Biomedical and Biotechnological Sciences, University of Catania, Catania, Sicily, Italy; ^3^Department of Clinical Neuroscience, Karolinska Institutet (KI), Stockholm, Sweden; ^4^Ocular Oncology and Pathology Services, St. Erik Eye Hospital, Stockholm, Sweden

**Keywords:** uveal melanoma (UM), choroidal melanoma, ophthalmology, oncology, pathology, melanoma, prognosis, metastasis

Uveal melanoma (UM) is the most common primary intraocular malignancy in adults, with a high propensity for metastasis and, historically, a lack of effective treatment options for late-stage disease. However, recent advancements in research and clinic have shed light on promising therapeutic approaches for UM. The Research Topic titled “*Uveal Melanoma: From Lab Bench to Clinic - New Therapeutic Advances*,” published by Frontiers, encompasses a collection of six articles that provide valuable insights into the understanding and management of this aggressive cancer.

One of the articles, by Wang et al. introduces a novel immune-related gene signature for prognosis and the tumor microenvironment in patients with UM. The authors employed single-cell and bulk sequencing data to construct an immune-related prognostic signature, which proved to be a strong predictive factor for overall survival. This research contributes to our understanding of the molecular and immune classification of UM and holds promise for future cancer immunotherapy strategies.

Another study, conducted by Medek et al. focuses on the early treatment and toxicity outcomes for UM using custom-loaded eye plaques. The authors compared the use of partially loaded Collaborative Ocular Melanoma Study (COMS) plaques to fully loaded plaques and there were no melanoma-related deaths, local recurrences or metastases in either group. Patients treated with custom-loaded plaques had significantly lower rates of visual loss. This finding suggests that custom-loaded plaques could improve treatment accuracy and minimize side effects in patients with small, posterior UM.

Hu et al. present a study exploring the development and validation of an immunogenic cell death (ICD)-related signature for predicting prognosis and the immune landscape of UM. By analyzing ICD-related gene expression, the researchers identified a 5-gene risk signature that serves as a novel prognostic biomarker. Their findings highlight the potential of ICD as an immunotherapeutic strategy for UM treatment.

In another article, Slater et al. investigate the role of cysteinyl leukotriene receptor 1 (CysLT1) and 1,4-dihydroxy quininib in UM by using preclinical *in vivo* orthotopic xenograft models and *ex vivo* patient samples. The authors found that high CysLT1 expression was associated with worse overall survival in an independent primary UM patient cohort. They also demonstrate that treatment of UM explants with 1,4-dihydroxy quininib modifies the secretion of inflammatory factors. Moreover, in a cell line-derived metastatic UM xenograft model, 1,4-dihydroxy quininib also affected ATP5F1B expression (a marker of oxidative phosphorylation), which is a poor prognostic indicator. These results highlight the diagnostic potential of CysLT1 and *ATP5F1B* and suggest that 1,4-dihydroxy quininib is a candidate for treatment of metastatic UM.

Chen et al. contribute to the Research Topic by identifying a prognostic model using cuproptosis-related genes (CRGs) in UM. Their analysis of the Cancer Genome Atlas (TCGA) database led to the construction of a prognostic gene model that showed a significant correlation with patient outcomes. This sheds new light on the role of CRGs as strong biomarkers in cancer progression and hints at their potential as therapeutic targets. Specifically, the study provides insights into the role of cuproptosis in UM and offers a potential prognostic tool for predicting patient survival.

Finally, Gill et al. present a comprehensive nationwide survey of UM in Sweden over five decades. This study examines changes in incidence rates, patient age and tumor size at diagnosis, treatment practices, and survival outcomes of 3,898 patients diagnosed with UM between 1960 and 2009. Crude and age-standardized incidence rates remained stable throughout the period. However, disease-specific survival has improved, likely due to decreasing tumor size at the time of diagnosis. These results emphasize the importance of early diagnosis and treatment in enhancing patient outcomes.

Collectively, these six articles contribute with advancements in our understanding and management of UM. They cover a wide range of topics, including immune-related prognostic signatures, customized treatment approaches, immunotherapeutic strategies, and the identification of prognostic biomarkers. Crucial insights into molecular mechanisms, treatment modalities, and prognostic factors are provided. In conclusion, the Research Topic “*Uveal melanoma: from lab bench to clinic - new therapeutic advances*” sheds light on the latest discoveries and advancements in UM research.

The findings contribute to the ongoing efforts to develop more effective and targeted therapies for UM, a challenging malignancy with limited treatment options. By unraveling the complex biology of UM and exploring innovative therapeutic avenues, researchers are paving the way for improved patient care and outcomes.

The Research Topic underscores the importance of interdisciplinary collaboration between basic scientists, clinicians, and researchers to translate laboratory discoveries into clinical applications. It also highlights the significance of leveraging advanced technologies and genomic profiling to enhance our understanding of UM and identify novel therapeutic targets.

Moving forward, it is crucial to continue building upon these advancements and conduct further studies to validate and refine the findings presented in this Research Topic. Collaborative efforts across research institutions and international networks will be instrumental in accelerating progress and driving innovation in the field of UM.

Overall, the Research Topic “*Uveal melanoma: from lab bench to clinic - new therapeutic advances*” provides a comprehensive overview of the recent breakthroughs in UM research and offers valuable insights that can guide future research endeavors and clinical practice. It offers a small but important step toward improving the prognosis and quality of life for patients affected by this aggressive ocular malignancy. We are most thankful to all authors, editors, reviewers, and patients that contributed to this Research Topic!

## Author contributions

All authors listed have made a substantial, direct, and intellectual contribution to the work and approved it for publication.

